# Intermolecular correlations are necessary to explain diffuse scattering from protein crystals

**DOI:** 10.1107/S2052252518001124

**Published:** 2018-02-21

**Authors:** Ariana Peck, Frédéric Poitevin, Thomas J. Lane

**Affiliations:** a Department of Biochemistry, Stanford University, Stanford, CA 94305, USA; b Department of Structural Biology, Stanford University, Stanford, CA 94305, USA; c Stanford PULSE Institute, SLAC National Accelerator Laboratory, Menlo Park, CA 94025, USA; d Bioscience Division and Linac Coherent Light Source, SLAC National Accelerator Laboratory, Menlo Park, CA 94025, USA

**Keywords:** diffuse scattering, intermolecular correlations

## Abstract

Conformational changes drive protein function, including catalysis, allostery and signaling. X-ray diffuse scattering from protein crystals has frequently been cited as a probe of these correlated motions, with significant potential to advance our understanding of biological dynamics. However, recent work has challenged this prevailing view, suggesting instead that diffuse scattering primarily originates from rigid-body motions and could therefore be applied to improve structure determination. To investigate the nature of the disorder giving rise to diffuse scattering, and thus the potential applications of this signal, a diverse repertoire of disorder models was assessed for its ability to reproduce the diffuse signal reconstructed from three protein crystals. This comparison revealed that multiple models of intramolecular conformational dynamics, including ensemble models inferred from the Bragg data, could not explain the signal. Models of rigid-body or short-range liquid-like motions, in which dynamics are confined to the biological unit, showed modest agreement with the diffuse maps, but were unable to reproduce experimental features indicative of long-range correlations. Extending a model of liquid-like motions to include disorder across neighboring proteins in the crystal significantly improved agreement with all three systems and highlighted the contribution of intermolecular correlations to the observed signal. These findings anticipate a need to account for intermolecular disorder in order to advance the interpretation of diffuse scattering to either extract biological motions or aid structural inference.

## Abstract 2

A comprehensive comparison of disorder models indicates that intermolecular correlations must be accounted for to explain the diffuse scattering observed from three protein crystals.

## 1. Introduction   

X-ray diffraction images from macromolecular crystals frequently exhibit a diffuse background between and beneath the Bragg peaks (Wall, Adams *et al.*, 2014 ▸; Welberry & Weber, 2016 ▸). In contrast to the Bragg reflections, which arise from coherent diffraction across the crystal, this diffuse signal results from disorder-induced incoherent diffraction. The uncorrelated disorder of solvent and macromolecular atoms yields a trivial diffuse scattering pattern that is radially symmetric (Moore, 2009 ▸). On the other hand, correlated disorder produces anisotropic diffuse scattering features whose spacing and intensity in reciprocal space are respectively determined by the length scale and amplitudes of the correlated atomic displacements involved (Benoit & Doucet, 1995 ▸).

Correlated displacements in macromolecules underlie many biological functions, such as allostery, signaling and enzyme catalysis. However, methods for directly measuring such motions with high spatial resolution are rare. Diffuse scattering has routinely been cited as one such method that could provide unique insights into the collective motions responsible for biological functions (Wall, Adams *et al.*, 2014 ▸; Benoit & Doucet, 1995 ▸). Indeed, this has motivated the majority of studies of diffuse scattering from macromolecular crystals (Wall *et al.*, 1997 ▸; Héry *et al.*, 1998 ▸; Kolatkar *et al.*, 1994 ▸; Chacko & Phillips, 1992 ▸; Meinhold & Smith, 2005*a* ▸,*b* ▸, 2007 ▸; Wall, Van Benschoten *et al.*, 2014 ▸; Van Benschoten *et al.*, 2016 ▸), despite the technical challenges of measuring and the computational cost of modeling this signal (Wall, Adams *et al.*, 2014 ▸).

Recently, the application of diffuse scattering to complement and improve (static) structure inference from crystal diffraction has also been proposed. Chapman and colleagues have suggested that in cases where measurable diffuse scattering extends to higher resolution than the Bragg data, it may be employed for structural inference at that higher resolution (Ayyer *et al.*, 2016 ▸). Further, the ability to oversample the diffraction pattern by measuring the continuous diffuse signal raises the possibility of solving the phase problem directly, without resorting to anomalous or isomorphic methods. This approach, however, assumes that diffuse scattering primarily originates from specific types of disorder, such as rigid-body motions, which are unlikely to inform on biological function.

Identifying the physical origins of diffuse scattering, and thus its potential for probing biological motions or advancing methods, remains a challenge for the field. Many types of disorder involve small motions that can be conveniently described by a matrix, the elements of which give the covariation between the displacements of any two atoms from their mean positions. Such a covariance matrix can directly, but not uniquely, predict diffuse scattering. If the covariance matrix could be inferred directly from experiment, the diffuse signal could be analyzed to determine which regions of the macromolecule move together. There is a fundamental problem with direct inference, however: while the number of observed independent variables is quite large (say *V* voxels for a given unit-cell volume, assuming that the maximum resolution of diffraction is fixed), the number of unknowns is even larger (of the order of *V* ^2^ matrix elements, one for each atom pair, assuming that the number of atoms scales linearly with volume). Thus, to infer a covariance matrix one must make simplifying assumptions about the nature of protein motions: a parsimonious model for the protein physics is required.

In this work, we analyze the parsimonious models that have been suggested previously (reviewed in Meisburger *et al.*, 2017 ▸) and variations of these models to critically examine the types of disorder underlying the diffuse scattering observed in a range of systems. The three protein systems analyzed here represent both diverse crystalline properties and biological functions: cyclophilin A (CypA), a monomeric proline isomerase (Van Benschoten *et al.*, 2016 ▸); WrpA, a flavodoxin-like protein (Peck *et al.*, 2016 ▸); and a dimeric enzyme, alkaline phosphatase (AP), bound to its transition-state analog (Herrou *et al.*, 2016 ▸). Despite this diversity, we found that multiple models of intramolecular conformational dynamics were unable to explain the observed diffuse scattering in all three systems. By contrast, models of simpler dynamics, including rigid-body and liquid-like motions, consistently showed modest correlation with the experimental signal when correlations were confined to the biological unit. The conventional form of the liquid-like motions model, in which correlations span neighboring protein molecules in the crystal, significantly improved the agreement both quantitatively and qualitatively, but still did not fully account for the observed signal. This comprehensive comparison both reconciles opposing viewpoints about the principal physical origins of diffuse scattering and specifically identifies intermolecular correlations as a critical component of the underlying disorder. Further, these findings anticipate that deconvolving the intermolecular contribution from the signal will be required to enable the future application of diffuse scattering to probe biological motions or to improve static structure inference.

## 2. Methods   

### 2.1. Reconstruction of three-dimensional diffuse scattering maps   

For each experimental data set, the Bragg reflections were indexed by *XDS* (Kabsch, 2010*b* ▸). Refined parameters from *XDS* were then used to determine the reciprocal-space co­ordinates of each pixel in every diffraction image. Measured intensities were corrected for polarization of the X-ray beam (Hura *et al.*, 2000 ▸) and the difference in solid angle subtended by pixels at different scattering angles (Wall, 2009 ▸). Per-image scale factors from *XDS* were applied to correct for differences in overall intensity across the rotation range (Kabsch, 2010*b* ▸). For data sets collected on a PILATUS detector, parallax broadening was also accounted for, as implemented in *DIALS* (Waterman *et al.*, 2016 ▸; Winter *et al.*, 2018 ▸). No Lorentz correction was applied, as pixel intensities were averaged rather than integrated, and the diffuse features were observed to vary gradually in reciprocal space. Under these conditions, the volume of reciprocal space integrated by each pixel only needs to be corrected for the solid angle subtended, and not the arc length that the pixel traverses owing to rotation of the crystal (Boysen & Adlhart, 1987 ▸).

After correcting for geometrical distortions, Bragg peaks were removed by implementing the spot-prediction algorithm described in Kabsch (2010*a* ▸). Pixels predicted to be spanned by a Bragg reflection were masked if their intensity exceeded three standard deviations above the mean intensity of neighboring pixels outside the predicted reflection region in a 30 × 30 pixel window centered on the reflection. Prior studies have replaced masked pixel intensities by the intensities from adjoining pixels (Polikanov & Moore, 2015 ▸) or pre-filtered images (Ayyer *et al.*, 2016 ▸), but the strategy of masking without replacement was found to maintain an adequate signal-to-noise ratio for map voxels that coincided with Miller indices (Supplementary Fig. S1, solid and dashed lines). To ensure the complete removal of Bragg contaminants, an additional step of masking was performed to eliminate pixels whose intensities exceeded the median radial intensity by more than five times the median absolute deviation of that resolution shell. In the case of WrpA, pixels were additionally masked if they fell within a region contaminated by scattering from the cryo-loop and exceeded the median radial intensity by more than 2.5 times the median absolute deviation of the resolution shell. Supplementary Fig. S2 shows the results of this Bragg masking procedure for a representative image from CypA.

The radial intensity profiles of the images were then compared for uniformity to ensure that independent measurements of equivalent positions in reciprocal space were on the same scale prior to merging. As noted above, variation in the total protein scattering across the rotation range, owing to fluctuations in the illuminated crystal volume and incident beam intensity for instance, was corrected by applying *XDS* scale factors used to similarly normalize the Bragg intensities. We assumed that large, radially symmetric changes in the measured scattering as the crystal is rotated originate from sources of noncrystalline scattering instead, such as solvent or coating oil. Based on this assumption, the largest variance components between the radial intensity profiles were determined and removed from each image. This approach was validated by comparing symmetry-equivalent positions in the corrected maps, which were found to be consistent. This observation supports our initial assumption that large-variance components were indeed caused by scattering from sources other than the protein crystal.

In the case of CypA, a radially symmetric peak at |**q**| = 1.3 Å^−1^ was variably observed, consistent with scattering from the Paratone oil in which the crystal was coated prior to data collection. In images without visible Paratone contamination, the experimental radial intensity profiles approximately followed a second-degree polynomial in the neighboring region of 0.63 < |**q**| < 1.88 Å^−1^. Based on this observation, the radial intensity profile for each image was fitted in this region of |**q**| to the sum of a second-degree polynomial and a scaled Paratone profile: *a*|**q**|^2^ + *b*|**q**| + *c* + *mI*_ref,Paratone_(|**q**| − *q*_0_). The reference Paratone profile, *I*_ref,Paratone_(|**q**|), was derived from the computed structure factors for noncrystalline Paratone N (Holton, 2016 ▸). Fitted parameters associated with the Paratone profile were a multiplicative scale factor, *m*, and an offset in |**q**|, *q*_0_. The latter parameter accounts for anisotropy in the distribution of the Paratone coating the crystal; optimized values of *q*_0_ were small, with a mean of *q*_0_ = 0.007 Å^−1^ across the 360 images in the CypA data set. The resulting Paratone profile was then subtracted from each image. Principal component analysis was performed to remove residual radial variance from the corrected images, and a background profile for each image was generated from the sum of the first two principal components, which were scaled by their associated eigenvalues and projected onto the radial intensity profile of the image. The intensity correction for each pixel was then estimated by linear interpolation from these background profiles and subtracted from each image. In the case of WrpA, the first principal component was similarly used to remove variance in the radial intensity distributions across the rotation range. No further corrections were applied to the AP diffraction images.

Diffuse scattering maps were constructed as three-dimensional grids in reciprocal space whose nodes oversample Miller indices by a factor of three along each lattice direction. Corrected intensities were binned into voxels centered on these nodes, and the mean pixel intensity of each voxel was used to estimate the intensity at each node. The signal-to-noise ratio was estimated as the mean divided by the standard deviation of the intensities binned into each voxel and is shown across resolution shells in Supplementary Fig. S1. Maps were symmetrized by averaging the intensities of Laue- and Friedel-equivalent voxels, followed by subtraction of the interpolated radial intensity. If symmetrization is justified, this order of operations will provide a better estimate of the intensities used to compute the radial average profile; if not, it will introduce bias. However, reversing the symmetrization and radial average subtraction steps was not observed to affect the results, with all correlation coefficients (CCs) observed to be within 0.01 of their prior values. A constant value was then added uniformly to each symmetrized, radial average-subtracted map to ensure that all intensities were positive. The code used to generate the maps is available at https://github.com/apeck12/diffuse.

### 2.2. Bragg data processing   

Refined structural models from the analysis of the Bragg component of these data sets have previously been reported (Van Benschoten *et al.*, 2016 ▸; Peck *et al.*, 2016 ▸; Herrou *et al.*, 2016 ▸). However, because the diffuse maps were generated using refined parameters and a modified version of the spot-prediction algorithm from *XDS*, the Bragg data were reprocessed to maximize consistency between the treatment of the Bragg and diffuse signals. The Bragg data were indexed, integrated and scaled with *XDS* (Kabsch, 2010*b* ▸); statistics are shown in Table 1 ▸. For CypA, molecular replacement was performed with *Phaser* (McCoy *et al.*, 2007 ▸) using PDB entry 2cpl (Ke, 1992 ▸) as a search model. This was followed by five macrocycles of refinement in *PHENIX* (Adams *et al.*, 2010 ▸) as previously described (Van Benschoten *et al.*, 2016 ▸). For AP, molecular replacement was performed with *Phaser* using wild-type AP (PDB entry 3tg0; Bobyr *et al.*, 2012 ▸) stripped of non­protein atoms as the search model. As in Peck *et al.* (2016 ▸), zinc ions at full occupancy and a tungstate ion and water molecules at partial occupancy were manually modeled into the residual electron density in each active site. Automated refinement was performed using *REFMAC*5 (Murshudov *et al.*, 2011 ▸). For WrpA, molecular replacement was performed with *Phaser* using PDB entry 5f51 as a search model (Herrou *et al.*, 2016 ▸). This was followed by alternating rounds of manual refinement in *Coot* (Emsley *et al.*, 2010 ▸) to model a sulfate ion and water molecules, and automated refinement in *Phenix*. The *R*_work_/*R*_free_ values of the final refined models were similar to those deposited previously (Van Benschoten *et al.*, 2016 ▸; Peck *et al.*, 2016 ▸; Herrou *et al.*, 2016 ▸). Diffraction images for the CypA, AP and WrpA data sets are available in the SBGrid Data Bank, with accession Nos. 68 (Fraser, 2015 ▸), 456 (Peck *et al.*, 2017 ▸) and 203 (Herrou & Crosson, 2015 ▸), respectively.

### 2.3. Diffuse scattering predictions from real-space models of disorder   

Experimental diffuse scattering maps were compared with the following disorder models.

(i) A Gaussian elastic network model, a commonly used normal-mode decomposition of the protein motions based on the structure. Normal modes were computed using a uniform spring constant for all atom pairs within a given distance (Bray *et al.*, 2011 ▸), and the predicted interatomic correlations were renormalized by the *B* factors from the refined models of the Bragg data.
(ii) Conformational ensemble models, which model configurational disorder as a discrete set of probability-weighted states (Guinier, 1963 ▸). Conformational states were inferred by analyzing the crystal electron density from the Bragg data.
(iii) Rigid-body rotations, in which the atoms in an asymmetric unit rotate as a unit around a random, isotropically oriented axis with a normally distributed rotation angle (Moore, 2009 ▸).
(iv) Rigid-body translations, in which the atoms in an asymmetric unit translate as a unit. Translations sample an isotropic Gaussian distribution (Moore, 2009 ▸; Ayyer *et al.*, 2016 ▸).
(v) Liquid-like motions, in which correlations between atoms decay exponentially as a function of interatomic distance (Wall *et al.*, 1997 ▸; Clarage *et al.*, 1992 ▸). Two forms of this model were considered: a variant in which correlations were confined within the boundaries of the asymmetric unit and the conventional form of this model, in which correlations extend between neighboring protein molecules, thereby crossing asymmetric unit and unit-cell boundaries.

For all models except for the conventional liquid-like motions model, correlations were assumed to be confined within the boundaries of the asymmetric unit, with no coherence between neighboring molecules in the crystal lattice. For the systems considered here, the chosen asymmetric unit contained a single copy of the biological unit.

Diffuse scattering maps were simulated using *Thor* (Lane, 2017 ▸), a software package for simulating and analyzing X-ray scattering experiments. For consistency with the experimental maps, the average radial intensity was subtracted from the predicted maps. Best-fit parameters for each model were determined by scanning over the disorder parameter(s) to maximize the CC with the experimental map. Agreement was assessed by the CC between the predicted and experimental maps, with each voxel downweighted by its multiplicity. For visual comparisons, a multiplicative scale factor and constant platform were applied to place the predicted maps on the same intensity scale as the experimental maps, unless otherwise noted. Disorder models are described in more mathematical detail in the Supporting Information.

## 3. Results   

### 3.1. The experimental maps exhibit Laue symmetry and significant anisotropic features   

**Figure 1:**
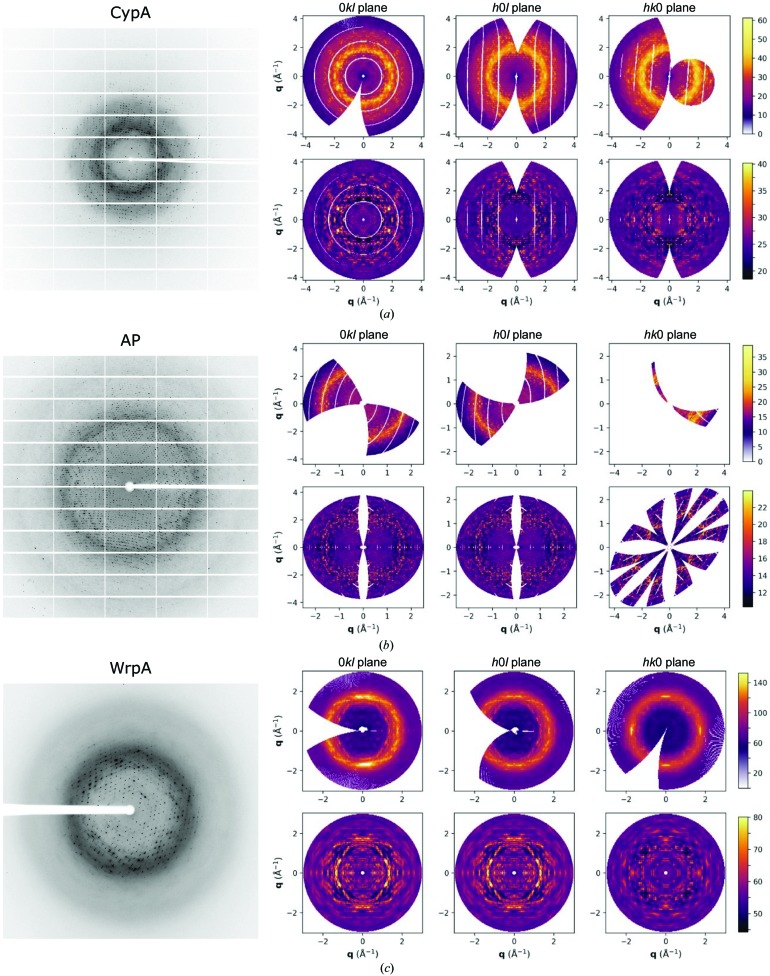
Reciprocal-space maps from experimental diffuse scattering. The diffuse scattering in diffraction images collected for (*a*) CypA, (*b*) AP and (*c*) WrpA was reconstructed into a reciprocal-space map for each system. These maps take the form of three-dimensional grids that are oversampled by a factor of three relative to the Miller indices along each lattice axis. Left: an example diffraction image from each data set. Right: central slices through reciprocal space are visualized for each unsymmetrized map in the top panels. The lower panels show these slices after symmetrization of Friedel- and Laue-equivalent voxels, followed by subtraction of the average radial intensity profile to highlight anisotropic features. For the symmetrized maps, the color scales do not span the entire range of voxel intensities; this saturates a subset of voxels but improves the overall contrast.

We analyzed three crystallographic data sets collected by the rotation method for which diffuse scattering was visible in the raw diffraction images (Fig. 1 ▸, left). The Bragg data were separated and processed using standard protocols, yielding refined structural models similar to those published previously (Van Benschoten *et al.*, 2016 ▸; Peck *et al.*, 2016 ▸; Herrou *et al.*, 2016 ▸). The diffuse scattering was isolated and processed to generate three-dimensional maps in reciprocal space (Fig. 1 ▸, upper right panels). The maps oversample the diffuse scattering signal along each lattice direction by a factor of three relative to the Miller indices, which enables these maps to resolve correlations that extend across multiple unit cells.

Overall statistics for the diffuse scattering maps are shown in Table 1 ▸ and statistics by resolution shell are shown in Supplementary Fig. S1. The intensities of voxels related by Friedel’s law and Laue symmetry showed significant correlation in all cases, supporting symmetrization of the maps by averaging the intensities of these symmetry-equivalent voxels. Currently there is no established convention for determining when the symmetrization of diffuse maps is justified, so we considered the CC between symmetry-related voxels to be significant based on the threshold value of CC_1/2_ used to determine resolution cutoffs for the Bragg data (Karplus & Diederichs, 2012 ▸). Although these cases are not precisely analogous, this enhances the consistency between Bragg and diffuse data-processing techniques. To remove the intensity contributions from uncorrelated disorder, which includes solvent and air scattering in addition to uncorrelated protein disorder, the average radial intensity was subtracted from each map. The resulting maps were characterized by significant anisotropic features indicative of correlated disorder (Fig. 1 ▸, lower right panels).

### 3.2. Models of conformational dynamics do not correlate with the experimental maps   

**Figure 2:**
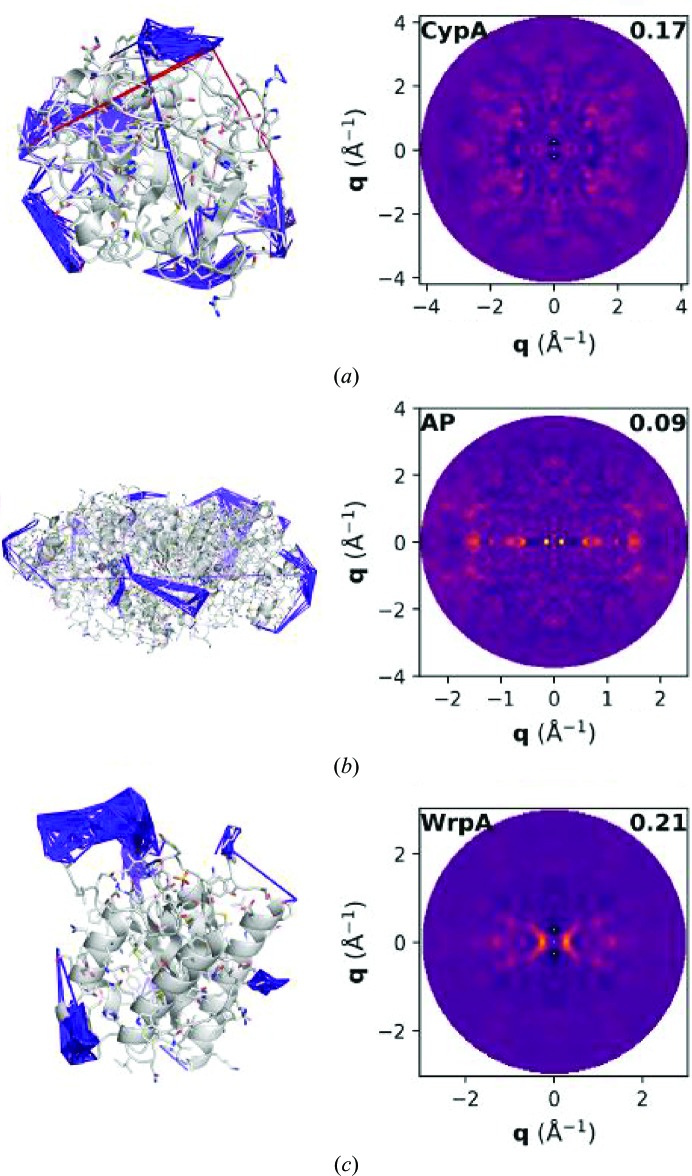
Elastic network models of Gaussian disorder. The highest and lowest magnitude entries in the covariance matrix are overlaid as blue and red cylinders, respectively, on the refined atomic coordinates for (*a*) CypA, (*b*) AP and (*c*) WrpA. The 0*kl* slices of the predicted diffuse scattering maps are shown on the right, with the overall CC noted in black. The color scales differ from Fig. 1 ▸ to enhance visualization of the features.

Models of intramolecular disorder that predict idiosyncratic configurational dynamics, the type of motions that are most likely to be related to biological function, were assessed for their ability to reproduce the experimental signal. A general class of these models assumes that interatomic displacements are small and sample a Gaussian distribution, and can thus be described by a covariance matrix. Here, covariance matrices were predicted from normal-mode analysis of each protein structure in torsion-angle space, using a standard form of the elastic network model that has been validated against Bragg-derived crystal structures (Bray *et al.*, 2011 ▸). Interatomic covariances were renormalized by the refined *B* factors such that the predicted amplitudes of motion were consistent with the structural models of the Bragg data. These elastic network models predicted distributions of strongly covarying atom pairs that were non-uniform and often spatially localized in the protein (Fig. 2 ▸, left, and Supplementary Fig. S3). For all three systems, the diffuse scattering predicted by these network models was unable to reproduce the observed signal, which was apparent both in the low CC and by visual comparison of the predicted and experimental 0*kl* planes (Fig. 2 ▸).

**Figure 3:**
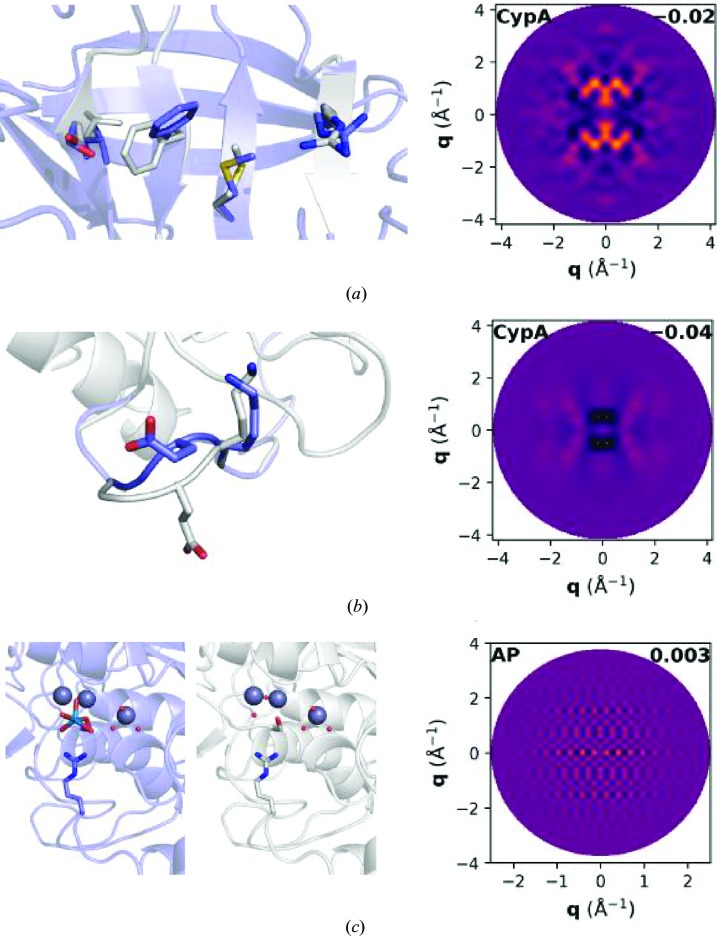
Ensemble models inferred from the Bragg data. (*a*) Multi-conformer modeling of the CypA electron-density map predicts a minor population of alternate conformers (purple) that radiate from the active site. (*b*) CypA data sets collected at 180 K and below show two loop conformations between residues 79 and 83; the visualized loop conformations are those modeled in the 100 K data set. Above 180 K, the conformation shown in white is not populated. (*c*) Model of AP assuming that one active site is tungstate-bound (left) while the other is occupied by water molecules that coordinate the active-site metal ions (right). The 0*kl* plane of the predicted map from each ensemble model is shown on the right, with a different color scale from Fig. 1 ▸ to enhance visual contrast. The overall CC between the experimental map and each predicted map is noted in black.

Non-Gaussian ensemble models inferred from the Bragg data were also evaluated to determine whether such conformational heterogeneity contributes measurably to the diffuse signal. In the case of CypA, multiconformer modeling of the electron-density map revealed a minor population of alternative rotamers for a series of residues that radiate from the active site (Fig. 3 ▸ *a*, left; van den Bedem *et al.*, 2009 ▸). This observation of a correlated rotameric switch is consistent with prior analysis of CypA crystal structures (van den Bedem *et al.*, 2009 ▸; Fraser *et al.*, 2009 ▸, 2011 ▸; Keedy *et al.*, 2015 ▸). Another ensemble was generated from the loop conformations populated by residues 79–83 in CypA crystals from which data were collected at or below 180 K (Fig. 3 ▸ *b*, left; Keedy *et al.*, 2015 ▸). Although only one of these conformations is populated in the data set analyzed here (which was collected at 273 K), this model offers a distinct example of a type of configurational disorder that is prevalent in proteins. A third ensemble model was suggested by the occupancy disorder observed in AP, for which the Bragg coordinates were refined with a half-occupied tungstate ion in each active site. However, the Bragg data cannot distinguish between this model of partial occupancy and a model of correlated occupancy in which only one AP monomer is tungstate-bound at a given time (Fig. 3 ▸ *c*, left). Whereas partial occupancy contributes to radially symmetric diffuse scattering, correlated occupancy yields anisotropic features.

Diffuse scattering maps were predicted for each two-state model by Guinier’s equation (Guinier, 1963 ▸); the 0*kl* planes are shown in Fig. 3 ▸. The predicted maps from the CypA ensemble models are distinct from one another, but both exhibit features spread over much broader regions of reciprocal space (owing to the short length scale of the disorder in real space) than observed experimentally (Fig. 1 ▸ *a* *versus* Figs. 3 ▸ *a* and 3 ▸ *b*). The map predicted by the correlated occupancy model shows a unique checkered pattern (Fig. 3 ▸ *c*), but these regular features are similarly larger than the features observed in the experimental map for AP (Fig. 1 ▸ *c*). Although diffuse scattering has been suggested as a route for validating conformational heterogeneity modeled during Bragg refinement (Moore, 2009 ▸; Wilson, 2013 ▸), these ensemble models do not appreciably account for the diffuse signal in these data sets (Table 2 ▸).

### 3.3. Short-range rigid-body and liquid-like motions models show modest agreement with the observed signal   

The inability of these elastic network and ensemble models to reproduce the experimental maps prompted us to evaluate other disorder models which predict simpler dynamics of rigid-body or liquid-like motions. As in the previous section, the models described below assume that correlations do not extend beyond the boundaries of individual asymmetric units. Additionally, the models evaluated in this section share the symmetrized molecular transform – specifically, the Fourier transform of the individual protein molecule, incoherently summed over its orientations in the unit cell – as their basis, which, as discussed below, raises the possibility of using the diffuse scattering signal for static structural inference.

**Figure 4:**
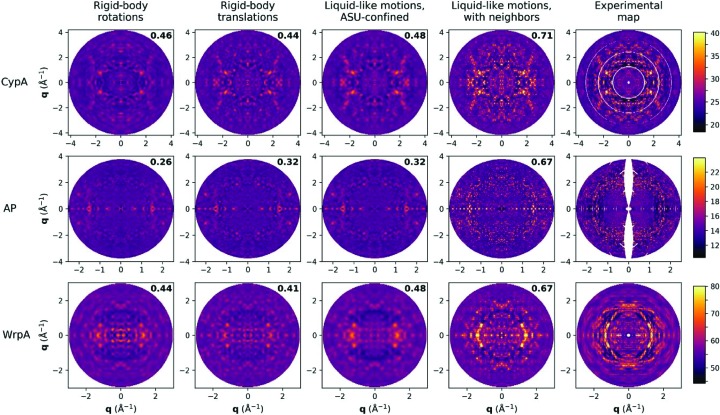
Comparison of models of rigid-body and liquid-like motions. For the indicated model, parameters that tune the disorder were fitted to maximize overall correlation with the experimental map. The predicted 0*kl* planes for the best-fit maps are shown, with the experimental 0*kl* planes displayed in the rightmost column for comparison. The overall CC between the experimental map and each predicted map is noted in black. As with Fig. 1, the color scales span a partial range of the voxel intensities to improve visual contrast.

The diffuse scattering predicted by rigid-body rotational disorder is related to the variance of an ensemble of rotated structure factors. Visually, this type of disorder has the effect of blurring features of the molecular transform in concentric shells of reciprocal space. An isotropic version of this model showed modest correlation with the CypA and WrpA maps (Fig. 4 ▸). For both maps, the best-fit values for the standard deviation of the angle of rotation were of the order of 2–3° (Table 3 ▸), consistent with a blurring effect that spans a few voxels of these reciprocal-space maps. The best-fit value for the AP map was smaller (0.9°), yielding minimal radial blurring that could be resolved by the coarseness of the map’s voxels, which along with the modest correlation suggested that rotational disorder was inconsistent with the observed signal. Relative to CypA and WrpA, AP has more crystal contacts that may inhibit this type of disorder. It is also possible that the finer slicing during the collection of the AP data minimized blurring, but radial blurring owing to data collection *versus* as a result of rotational disorder in the crystal cannot be distinguished by the isotropic model considered here.

By contrast, the diffuse scattering produced by rigid-body translational disorder is the molecular transform scaled by the Debye–Waller factor (Moore, 2009 ▸; Ayyer *et al.*, 2016 ▸). For all three maps, this disorder model showed nontrivial correlation with the experimental maps. Further, the best-fit values of the isotropic displacement parameter σ, which reports on the scale of displacement, were within twofold of the value predicted by the Bragg Wilson *B* factor (Fig. 4 ▸, Table 3 ▸), suggesting that the diffuse signal is consistent with scattered intensity missing in the Bragg data owing to disorder. The fit was modestly improved by imposing exponential decay on interatomic covariances, thereby switching from a rigid-body to a liquid-like description of correlated dynamics (Fig. 4 ▸). This model of asymmetric unit-confined liquid-like motions predicted similar though consistently smaller values for the isotropic displacement parameter and, in the case of CypA and WrpA, a correlation length roughly one third to one half the dimensions of the protein molecule (Table 3 ▸). In the case of AP, the best-fit correlation length spanned the longest dimension of the protein, consistent with the lack of improvement in CC: in the regime of correlation lengths longer than the protein unit, the diffuse scattering predictions of the liquid-like motions and rigid-body translational disorder models converge.

### 3.4. Speckles indicate long-range correlated disorder that crosses unit-cell boundaries   

**Figure 5:**
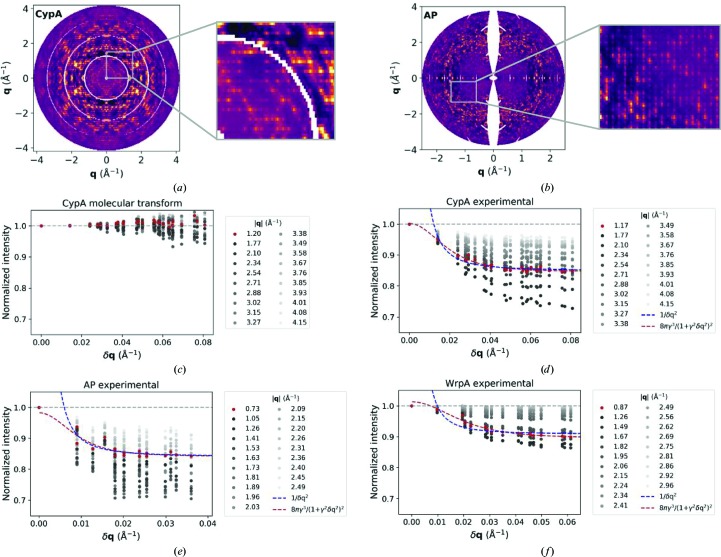
Speckled features of the experimental maps. The insets highlight the characteristic speckles for the 0*kl* planes of the (*a*) CypA and (*b*) AP maps. In (*c*)–(*f*), maps were reconstructed or computed with oversampling by a factor of five along each lattice direction relative to integral Miller indices. Data were binned into 20 resolution shells, and the median profile for the intensity as a function of δ**q**, the distance from reciprocal-lattice sites, is shown for each shell. Intensity profiles were normalized such that the intensity value for voxels that coincided with reciprocal-lattice sites (δ**q** = 0) was unity. For each experimental map, curves with a 1/δ**q** ^2^ (dashed blue) or a 8πγ^3^/(1 + γ^2^δ**q** ^2^)^2^ (dashed red) dependence, as predicted by the phonon and liquid-like motions models, respectively, were fitted to the intensity profile in the lowest resolution shell (red points). For comparison with models whose basis is the molecular transform, intensity profiles for the CypA molecular-transform map are shown in (*c*).

Of the models considered above, in no case does the correlation coefficient between the predicted and experimental map exceed 0.5 (Table 2 ▸, Supplementary Fig. S4). Visual inspection suggests that a feature that these models systematically fail to reproduce is the observed ‘speckles’: periodic spikes in intensity that appear superimposed on diffuse scattering features that span larger volumes in recip­rocal space (Figs. 5*a* ▸ and 5*b* ▸, insets). Such speckles arise from enhanced scattering at reciprocal-lattice positions, *i.e.* the estimated diffuse intensity underneath Bragg peaks, and have previously been noted in studies that analyzed the diffuse scattering signal at fractional Miller indices (Glover *et al.*, 1991 ▸; Polikanov & Moore, 2015 ▸; Meinhold *et al.*, 2007 ▸). Because the length scale of disorder in real space determines the spacing of diffuse features in reciprocal space, the need to oversample the diffuse signal relative to integral Miller indices to observe these speckles indicates that they arise from correlations that extend beyond the boundaries of a single unit cell. The models examined in prior sections assumed correlated disorder confined within asymmetric units and so were unable to generate this type of signal.

We therefore considered two models of disorder in which correlations extend across unit-cell boundaries to determine whether accounting for intermolecular correlations improved predictions of the total diffuse signal, including these speckled features. The first of these models is the traditional liquid-like motions model, in which the basis is the crystal rather than the molecular transform. For each system, this model of long-range liquid-like motions showed considerable agreement with the experimental signal, qualitatively reproducing the speckled features and quantitatively yielding the highest CC of the models considered here (Fig. 4 ▸, Table 2 ▸). This improved correlation is observed not just at the reciprocal-lattice sites where the speckles are centered, but also at the map voxels farthest from integral Miller indices (Supplementary Fig. S4*b*). The refined model parameters for the two liquid-like motions models were similar in most cases (Table 3 ▸), indicating that the consistent increase in CC between the short-range and long-range models resulted almost exclusively from taking into account correlations across neighbors in the crystal, which contribute to the diffuse signal throughout reciprocal space. Further support for this liquid-like motions model comes from a comparison of the predicted and experimental autocorrelation functions, from which correlation lengths in real space can be inferred. Peaks consistent with the unit-cell dimensions, and thus indicative of correlations extending across unit-cell boundaries, were observed in the autocorrelation function of each experimental map and the long-range liquid-like motions model. By contrast, models in which correlations were confined within the boundaries of the asymmetric unit did not reproduce these characteristic peaks (Supplementary Fig. S5).

An alternative model proposed to account for speckled features invokes acoustic lattice vibrations from phonon-induced inelastic scattering (Glover *et al.*, 1991 ▸; Polikanov & Moore, 2015 ▸; Meinhold *et al.*, 2007 ▸). One prediction of this model is that the diffuse intensity will decrease proportional to the square of the distance from reciprocal-lattice positions, δ**q**. Such a trend is absent from the molecular transform and its derivative models (Fig. 5 ▸ *c*), in which disorder is confined within the boundaries of asymmetric units. Although qualitatively the phonon model accounts for the observed halos around Bragg peaks, quantitatively the experimental fall-off in intensity differs from the 1/δ**q** ^2^ dependence predicted for single-phonon interactions and is better fitted by the dependence predicted by the liquid-like motions kernel (Figs. 5 ▸ *d*, 5 ▸ *e* and 5 ▸ *f*, dashed blue *versus* red; Glover *et al.*, 1991 ▸; Meinhold *et al.*, 2007 ▸). More complex phonon models, either from extending the spectrum to include optical modes or from accounting for multiple-phonon effects, are predicted to cause the diffuse intensity to vary more slowly with distance from reciprocal-lattice sites (Glover *et al.*, 1991 ▸). However, there is currently no robust method for predicting the diffuse scattering produced by phonons in macromolecular crystals. In the absence of such a method and an established procedure for simulating competing acoustic modes, let alone optical modes or the effects of multiple-phonon interactions, we cannot fully assess agreement with the phonon model *versus* other types of long-range disorder.

## 4. Discussion   

Here, we present a unified framework of the principal disorder models that have previously been used to interpret diffuse scattering, and compare their ability to reproduce the signal observed in three experimental data sets. Consistent with previous work, the above analysis finds that rigid-body and liquid-like motions models exhibit modest correlation with the experimental maps when correlated disorder is confined to the asymmetric unit (Ayyer *et al.*, 2016 ▸). Multiple models that predict more complex intramolecular dynamics were also considered, but showed minimal agreement with experiment (Table 2 ▸). Experimentally observed speckles did not fit the profile for phonon-induced lattice dynamics but, in agreement with prior results, could largely be reproduced by the conventional form of the liquid-like motions model, in which disorder extends across neighboring asymmetric units and thus unit-cell boundaries (Wall *et al.*, 1997 ▸; Doucet & Benoit, 1987 ▸; Van Benschoten *et al.*, 2016 ▸). None of the models assessed here fully explained the experimental signal in these data sets, which represent a range of crystallographic properties and biological functions. However, all three protein systems were globular proteins, and it is possible that distinct types of disorder underlie the diffuse scattering from crystals of membrane and fibrous proteins.

Past interest in diffuse scattering has primarily stemmed from the premise that these data probe dynamics related to biological function (Wall, Adams *et al.*, 2014 ▸; Héry *et al.*, 1998 ▸; Kolatkar *et al.*, 1994 ▸; Chacko & Phillips, 1992 ▸; Meinhold & Smith, 2005*a* ▸,*b* ▸, 2007 ▸; Wall, Van Benschoten *et al.*, 2014 ▸; Van Benschoten *et al.*, 2016 ▸). However, the experimental maps showed minimal correlation with the elastic network and ensemble models assessed here. These specific models represent a limited subspace of possible models of conformational dynamics that are consistent with the Bragg data, and it is likely that refining the parameters of the models could improve agreement with the diffuse signal. However, the observation of experimental features indicative of correlations that span neighboring molecules in the crystal cautions against the assumption that the dominant signal originates from the same protein motions that occur under physiological conditions, which these models attempt to capture. Models of disorder that account for both intermolecular and intramolecular correlations will thus be needed to resolve the contributions of each to the observed signal, a prerequisite in determining whether diffuse scattering is a useful method for studying dynamics associated with biological function.

On the other hand, the ability of rigid-body and liquid-like motions models to reproduce many experimental features suggests that diffuse scattering data could in some cases be useful for resolution extension or phase retrieval. These models share the molecular or crystal transform as their basis, and thus yield a scaled or blurred image of this transform in the diffuse scattering map. The general observation that the diffuse scattering does not *directly* reflect the molecular or crystal transform, but at the very least the convolution of the transform with some blurring function, must be better understood and taken into account.

Extracting the molecular-transform signal will be particularly challenging for maps that exhibit enhanced scattering at reciprocal-lattice sites, as observed here. The conventional liquid-like motions model, in which correlations are not confined to the asymmetric unit but rather extend between neighboring units in the crystal, best accounted for this feature. However, in the linear approximation, this model is a convolution of the disorder-free diffraction with a kernel that is the Fourier representation of the disorder. Thus, this model reports on the crystal transform, which is nonzero only at integral Miller indices. It does not contain information about the value of the molecular transform at fractional Miller indices. Iterative phase-retrieval algorithms, such as those recently used by Ayyer *et al.* (2016 ▸), require these non-integral oversampled measurements to uniquely determine un­measured phases (Sayre, 1952 ▸). Thus, the information present in the liquid-like motions model could in principle be employed for resolution extension, but not phase retrieval. However, the liquid-like motions model is approximate, and a more rigorous treatment of crystalline disorder may enable measurement of an oversampled molecular transform from the diffuse scattering. Despite this possibility, our results call into question the practice of directly using a diffuse scattering map for either resolution extension or iterative phasing in cases that exhibit enhanced scattering at reciprocal-lattice positions, a feature observed in all three systems we studied and one that we have no physical or theoretical grounds to mask or model separately from the remainder of the diffuse signal. Precisely how to deconvolve useful signals from such maps remains an open area of investigation.

The above analysis highlights the need for new models of diffuse scattering, either to interpret biologically relevant disorder or improve structure determination. If different sources of disorder are largely uncoupled, their contributions to the diffuse scattering will be approximately additive. However, the absence of coupling between distinct types of motions is not guaranteed, particularly in the context of a crystal lattice. Thus, challenges lie ahead both in jointly modeling distinct sources of disorder and in deconvolving weak signals from dominant features. The search space for such models is intractably large, so the number of acceptable free parameters and constraints will require careful treatment. Ideally, it would be possible to assert a model that is sophisticated enough to report interesting and idiosyncratic disorder in different systems (such as functional motions), but simple enough (*i.e.* with few independent parameters) to infer directly from the observed data.

An alternative route is detailed forward modeling, such as molecular dynamics, which has previously been used to analyze diffuse scattering (Faure *et al.*, 1994 ▸; Héry *et al.*, 1998 ▸; Wall, Van Benschoten *et al.*, 2014 ▸). Molecular dynamics concurrently simulates multiple types of disorder, but this method does not lend itself to refining the contributions of different kinds of disorder to fit experimental data. In the common case where such simulations do not satisfactorily reproduce experimental observations, it is challenging to modify them in a principled manner so that they do. Combined with the computational expense of these methods, it seems prudent to seek simple explanations and models for analyzing diffuse scattering before comparing with atomic simulation. The incisive test of any model will come from its predictive power: confirming that a specific physical perturbation of a crystal system results in the predicted change to the diffuse signal.

## 5. Conclusions   

Here, we investigated the physical origins of the diffuse scattering observed from three protein crystals. A comprehensive comparison of previously proposed models critically addressed the nature and length scale of the disorder underlying this signal. Multiple models of intramolecular conformational dynamics, including ensemble models inferred from the Bragg data, were unable to explain the observed diffuse scattering. While models of rigid-body and liquid-like motions of individual proteins consistently showed modest agreement with experiment, a model of extended liquid-like motions across the crystal achieved high correlations with the three data sets analyzed (CC ≃ 0.7). This analysis indicates that accounting for the intermolecular component of the disorder will be critical to successfully model this signal, which in turn is necessary to interpret diffuse scattering in order to either probe conformational dynamics or enhance static structure inference.

## Figures and Tables

**Table 1:** Data collection, model and map statistics

	CypA	AP	WrpA
Data-collection and Bragg statistics†			
Space group	*P*2_1_2_1_2_1_	*P*6_3_22	*P*4_2_22
Unit-cell parameters			
*a*, *b*, *c* (Å)	42.9, 52.4, 89.1	161.3, 161.3, 139.4	61.3, 61.3, 128.7
α, β, γ (°)	90.0, 90.0, 90.0	90.0, 90.0, 120.0	90.0, 90.0, 90.0
Wavelength (Å)	0.9795	0.9795	0.9787
Oscillation range (°)	0.5	0.15	1.0
Data-collection temperature (K)	273	100	100
Beam divergence SD (°)	0.0417	0.0285	0.0339
Mosaicity (°)	0.106	0.136	0.197
Resolution range (Å)	44.57–1.20 (1.24–1.20)	49.36–2.00 (2.07–2.00)	44.38–2.51 (2.60–2.51)
Multiplicity	5.8 (4.8)	6.3 (6.5)	12.7 (12.7)
Completeness (%)	93.6 (85.8)	94.0 (92.7)	99.6 (98.5)
〈*I*/σ(*I*)〉	11.3 (3.9)	26.2 (3.6)	25.5 (4.6)
*R*_merge_	0.113 (0.504)	0.045 (0.348)	0.061 (0.520)
*R*_meas_	0.123 (0.564)	0.049 (0.375)	0.063 (0.542)
CC_1/2_	0.989 (0.872)	1.000 (0.972)	0.999 (0.962)
Wilson *B* (Å^2^)	16.1	31.4	64.7
Detector	PILATUS 6M	PILATUS 6M	MAR Mosaic 300
SBGrid ID	68	456	203
Structure-refinement statistics			
Resolution range (Å)	44.57–1.20	49.36–2.00	44.37–2.50
No. of non-H atoms	1419	6731	1237
Average *B* factor (Å^2^)	21.4	46.2	77.0
Solvent content (%)	56	62	52
*R*_work_/*R*_free_	0.169/0.177	0.192/0.231	0.235/0.277
Diffuse scattering statistics			
Resolution range (Å)	93.4–1.5	75.2–2.5	69.3–2.1
CC_Friedel pairs_ ‡	0.979 (0.623)	0.954 (0.695)	0.988 (0.689)
CC_unsym, Friedel-sym_ ‡ §	0.996 (0.915)	0.994 (0.957)	0.997 (0.930)
CC_unsym, Laue-sym_ ‡ §	0.993 (0.862)	0.984 (0.887)	0.994 (0.827)
Completeness (%)	98.7	96.1	100.0

† Values in parentheses are for the highest resolution shell.

‡ For values in parentheses, the average radial intensity was subtracted prior to applying symmetry operations.

§ Correlation coefficient between the unsymmetrized map and the map after applying the indicated symmetry operations.

**Table 2:** Correlation coefficients between predicted and experimental maps

	Model				
	Rigid-body rotations	Rigid-body translations	Liquid-like motions†	Elastic network	Ensemble
CypA	0.46	0.44	0.48 | 0.71	0.17	−0.02, −0.04‡
AP	0.26	0.32	0.32 | 0.67	0.09	0.00§
WrpA	0.44	0.41	0.48 | 0.67	0.21	—

† Values before and after the vertical bar indicate the asymmetric unit-confined and ‘with neighbors’ models, respectively.

‡ Rotamer switch and disordered loop models, respectively.

§ Correlated occupancy model.

**Table 3:** Refined model parameters

	Model				
	Bragg Wilson *B* †	Rigid-body translations	Liquid-like motions‡		Rigid-body rotations
	σ (Å)	σ (Å)	σ (Å)	γ[Table-fn tfn10] (Å)	σ (°)
CypA	0.45	0.68	0.36 | 0.39[Table-fn tfn11]	18 | 18	2.9
AP	0.63	0.63	0.40 | 0.48	118 | 53	0.9
WrpA	0.90	1.05	0.54 | 0.61	15 | 18	3.4

† The Bragg σ was computed from the Wilson *B* factor.

‡ The values to the left and right of the vertical bar indicate the asymmetric unit-confined model and conventional model ‘with neighbors’, respectively.

§ The correlation length affects the volume in reciprocal space across which intensities of the crystal transform are blurred. Consequently, this parameter may be sensitive to how finely the diffuse signal is sampled. However, fitting the LLM to experimental maps constructed with oversampling by a factor of five relative to the Miller indices yielded similar values of γ, suggesting that the experimental features are sufficiently resolved with oversampling by a factor of three.

¶ Prior analysis of this data set found best-fit parameters of σ = 0.38 Å and γ = 7.1 Å for the model with neighbors (Van Benschoten *et al.*, 2016 ▸). However, in that study the diffuse signal was sampled at integral Miller indices and diffuse halos around Bragg peaks were suppressed.
